# Coil embolization for giant left anterior descending artery aneurysm with coronary pulmonary artery fistula in an elderly patient: a case report

**DOI:** 10.1093/ehjcr/ytag150

**Published:** 2026-03-05

**Authors:** Soichiro Shirabe, Michinari Hieda, Mitsuhiro Fukata, Seiichiro Gohara

**Affiliations:** Department of Hematology, Oncology, and Cardiovascular Medicine, Kyushu University Hospital, 3-1-1 Maidashi, Higashi-ku, Fukuoka 812-8582, Japan; Department of Hematology, Oncology, and Cardiovascular Medicine, Kyushu University Hospital, 3-1-1 Maidashi, Higashi-ku, Fukuoka 812-8582, Japan; Department of Cardiology and Nephrology, University of Occupational and Environmental Health Hospital, 1-1 Iseigaoka, Yahatanishi-ku, Kitakyushu, Fukuoka 807-8556, Japan; Department of Hematology, Oncology, and Cardiovascular Medicine, Kyushu University Hospital, 3-1-1 Maidashi, Higashi-ku, Fukuoka 812-8582, Japan; Department of Cardiology, Kyushu Central Hospital, 3-23-1 Shiobaru, Minami-ku, Fukuoka 815-8588, Japan

**Keywords:** Coronary artery aneurysm, Coronary pulmonary artery fistula, Giant aneurysm, Coil embolization, Balloon-assisted intervention, Elderly patient, Case report

## Abstract

**Background:**

Giant coronary artery aneurysms (CAAs), defined as dilatations exceeding 20 mm or four times the reference vessel diameter, are rare and risk rupture or thrombosis. Coronary pulmonary artery fistulas may lead to grow aneurysm formation via chronic high-flow shunting. Their coexistence is rare in elderly patients without prior Kawasaki disease.

**Case summary:**

An 83-year-old woman with hypertension, dyslipidaemia, and type 2 diabetes mellitus was referred for evaluation of an abnormal mediastinal contour. Chest computed tomography showed a 43 × 33 mm left anterior descending artery aneurysm with a fistulous to the pulmonary artery, grown from 30 × 20 mm over 10 years. Right heart cardiac catheterization showed no evidence of pulmonary artery hypertension with a mild shunt flow (Qp/Qs: 1.15). Despite being asymptomatic, the aneurysm's size and growth warranted intervention. Considering her age and comorbidities, transcatheter coil embolization was selected over surgical or stent-based therapies. Coils were deployed using intravascular ultrasound (IVUS) and a balloon-assisted technique, successfully occluding the aneurysm without compromising distal flow. Follow-up angiography at 6 months and echocardiography up to 3 years confirmed sustained occlusion.

**Discussion:**

This case highlights that even in asymptomatic elderly patients, large and expanding CAAs with fistulas warrant careful evaluation due to the risk of rupture. Transcatheter coil embolization provided a safe and effective treatment alternative to surgery, especially in high-risk anatomical and clinical settings. IVUS and balloon assistance were critical to procedural success, and long-term follow-up demonstrated sustained aneurysm exclusion.

Learning points

- Giant coronary aneurysms (>20 mm) with progressive growth should require intervention to prevent rupture, even if asymptomatic.
- Coil embolization, particularly with balloon assistance, is a safe and effective minimally invasive option in high-risk surgical patients.
- Avoiding stent placement in the native LAD artery reduced the risk of restenosis and allowed for single antiplatelet therapy in this high-bleeding risk patient.

## Introduction

Coronary artery aneurysms (CAAs) are defined as focal dilatations of a coronary artery exceeding 1.5 times the normal vessel diameter. Among them, those exceeding 20 mm or more than four times the reference diameter are termed ‘giant’ aneurysms. Giant CAAs are exceptionally rare, with an estimated prevalence between 0.02% and 0.2%.^1,2^ Coronary pulmonary artery fistulas (CPF), a congenital anomaly found in 0.1–0.2% of angiographic studies, can predispose to aneurysm formation through chronic left-to-right shunt.^3^ The coexistence of giant CAAs and CPFs is uncommon, especially in elderly patients without Kawasaki disease. No standard management approach exists, and clinical decisions must be individualized. In particular, the combination of a giant aneurysm with dual coronary-CPF is extremely rare, and current guidelines provide no specific recommendations regarding intervention in asymptomatic adults. Therefore, reporting such cases is important for refining clinical decision-making in anatomically complex CAAs/CPF disease.

## Summary figure

**Figure:**
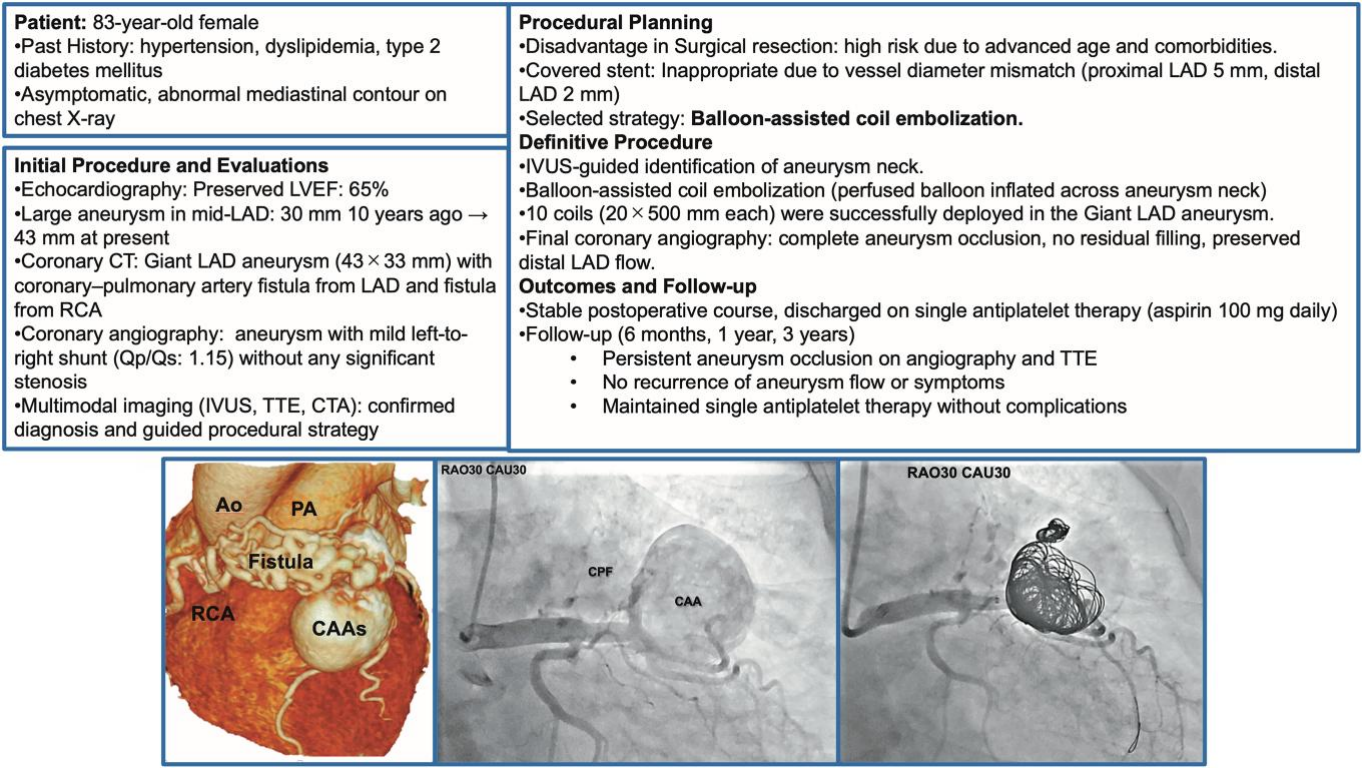


## Case presentation

**Figure 1:**
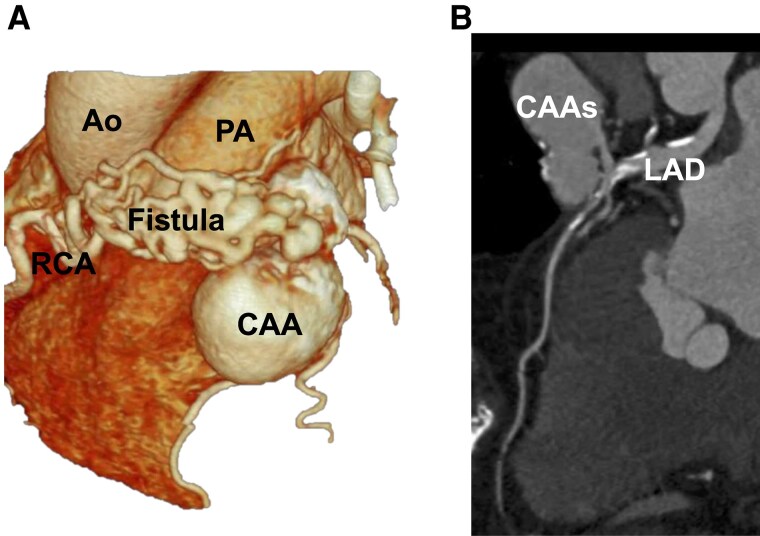
Pre-procedural coronary computed tomography (CT). (*A*) Three-dimensional coronary CT demonstrating a 43 × 33 mm saccular aneurysm arising from the mid-left anterior descending artery (LAD) with a fistulous connection to the pulmonary artery. The fistula from right coronary artery (RCA) to the pulmonary artery is also visible. (*B*) Multiplanar reconstruction (MPR) image clearly illustrating the anatomical relationship between the LAD and the coronary artery aneurysm (CAA), providing detailed visualization of the aneurysm neck and its connection to the LAD.

An 83-year-old woman with hypertension, dyslipidaemia, and type 2 diabetes mellitus presented with an abnormal mediastinal contour on a chest X-ray during a routine examination. She had no history of persistent fever, rash, red eyes, or lymph node swelling. There were no symptoms that suggested Kawasaki disease. Her vital signs were stable, with a blood pressure of 133/72 mmHg and a heart rate of 72 bpm. Physical examination revealed a continuous murmur at the left sternal border (Levine grade 2/6). Laboratory tests showed normal renal function, an LDL cholesterol level of 114 mg/dL, a B-type natriuretic peptide level of 7.3 pg/mL, and an elevated haemoglobin A1c of 8.8%, consistent with poorly controlled diabetes. White blood cell counts and CRP levels were in the normal range, suggesting no systemic inflammation. Her medications included teneligliptin 20 mg, amlodipine 5 mg, dapagliflozin 5 mg, and sacubitril/valsartan 200 mg (*Figure 1*).

**Figure 2:**
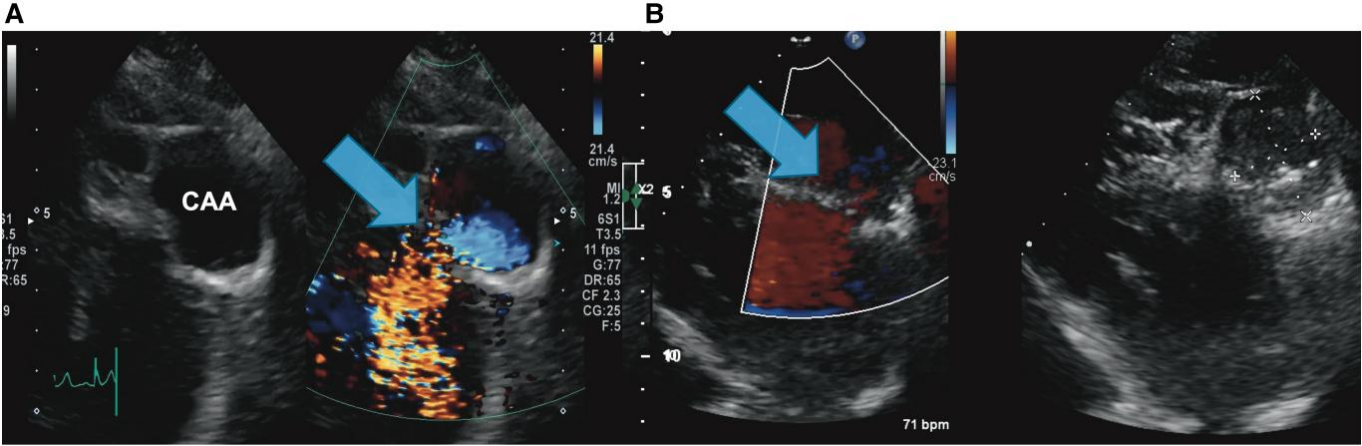
Transthoracic echocardiography (TTE) before and after the procedure. (*A*) Colour Doppler TTE before the procedure demonstrated flow from the LAD into the aneurysm sac. (*B*) Post-procedural TTE confirmed the complete cessation of flow within the aneurysm and presence of the coil. Blue arrows indicate the direction of flow into the coronary artery aneurysm (CAA).

**Figure 3:**
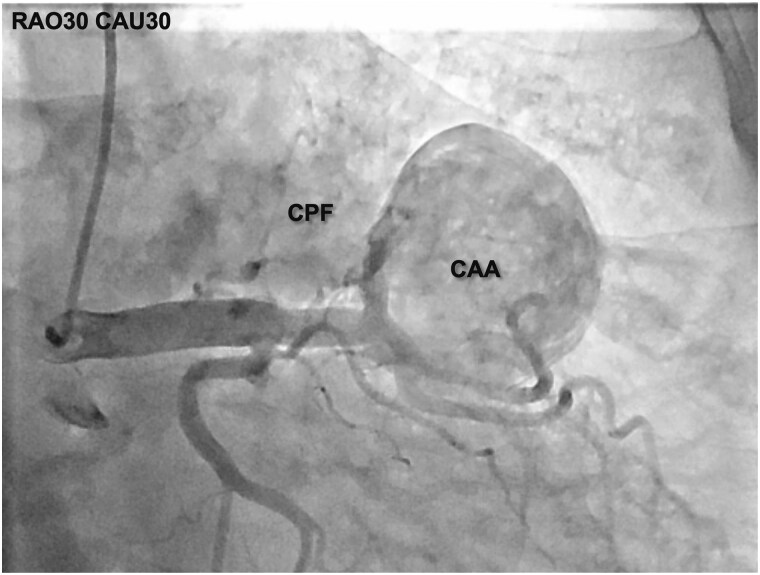
Pre-procedural coronary angiography. Angiogram showing contrast filling of a large LAD aneurysm with direct opacification of the pulmonary artery, consistent with a coronary pulmonary artery fistula. The aneurysm is saccular and demonstrates significant filling and outflow.

Transthoracic echocardiography (TTE) revealed a preserved left ventricular ejection fraction of 65%. Importantly, TTE clearly demonstrated a large saccular aneurysm in the left anterior descending artery (LAD), with visible flow from the LAD into the aneurysmal sac on colour Doppler imaging (*Figure 2*). Coronary computed tomography (CT) angiography confirmed a 43 × 33 mm saccular aneurysm arising from the mid-LAD with a fistulous connection to the main pulmonary artery, as well as an additional smaller fistulous connection from the right coronary artery (RCA) to the pulmonary artery (*Figures 1* and *3*). A retrospective review of TTE performed 10 years earlier revealed the same aneurysm at 30 × 20 mm, indicating significant growth.

Cardiac catheterization confirmed mild left-to-right shunt through the aneurysm and fistulous connections (Qp/Qs: 1.15, calculated using the Fick principle). There was no significant coronary artery stenosis. Myocardial perfusion scintigraphy showed no evidence of inducible ischaemia. Additionally, head magnetic resonance imaging and CT imaging ruled out other vascular aneurysms or vasculitis or arterial stenosis, further supporting the isolated nature of the coronary aneurysm.

**Figure 4:**
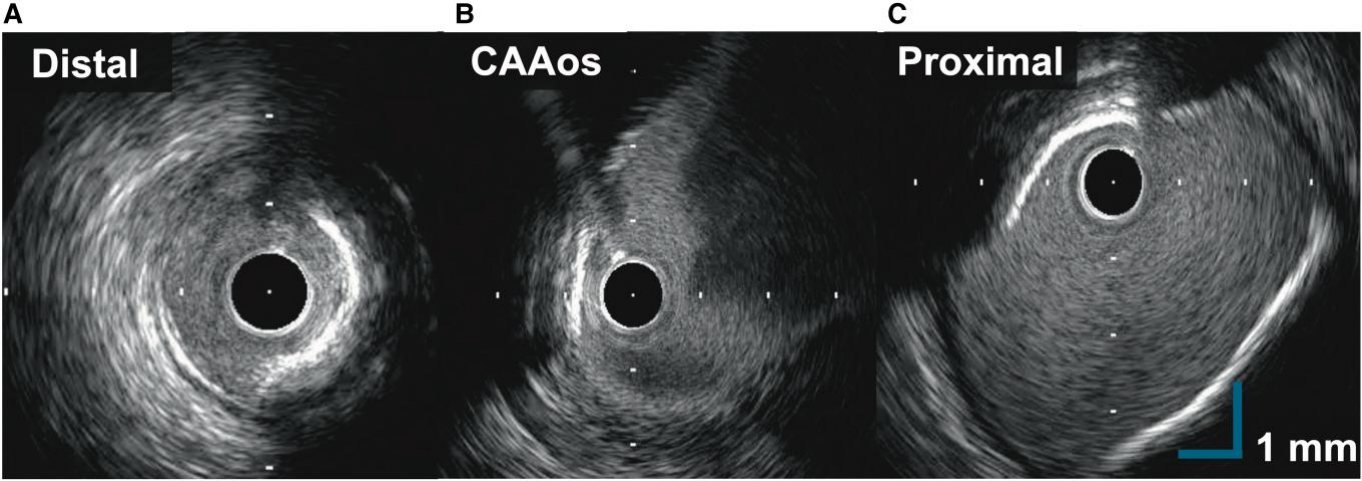
Intravascular ultrasound (IVUS) imaging of the aneurysm. IVUS image delineated the aneurysm neck and the adjacent LAD vessel wall. **(***A***)** IVUS of the distal LAD; (*B*) IVUS showing the aneurysm ostium from within the LAD; (*C*) IVUS of the proximal LAD. These images delineate the aneurysm neck and the adjacent LAD vessel wall. The entry point from the LAD into the aneurysm and vessel wall integrity is clearly visualized. The entry point from the LAD into the aneurysm and vessel wall integrity is clearly visualized.

Our heart team considered three options: (1) surgical resection with fistula ligation, (2) percutaneous covered stent placement, and (3) transcatheter coil embolization. Given the patient's advanced age and comorbidities, surgery was deemed high risk. Covered stent placement was considered inappropriate due to a marked discrepancy in vessel diameter between the proximal and distal landing zones. The proximal LAD measured approximately 5 mm, while the distal segment measured around 2 mm (*Figure 4*). This anatomical mismatch raised concerns about poor stent apposition and inadequate sealing. Eventually, the coil embolization was selected as the most appropriate option.

**Figure 5:**
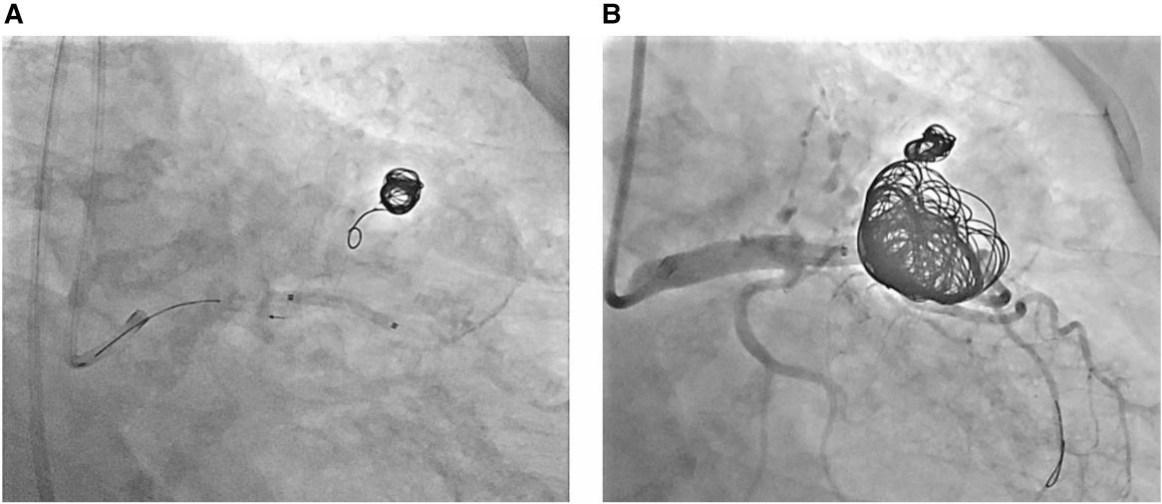
Coil embolization procedure. (*A*) Angiographic image during coil deployment under perfusion balloon inflation showing delivery of coils into the aneurysm while maintaining distal LAD flow. (*B*) Final angiogram demonstrating successful embolization, with complete cessation of flow into the coronary artery aneurysm and no residual contrast filling.

The intervention was performed using the right femoral artery approach. An 8 Fr Judkins Left (catheter) 3.5 guiding catheter was used to engage the left coronary system. A 0.014-inch guidewire was advanced into the distal LAD. Intravascular ultrasound (IVUS) was used to indicate the aneurysm neck (*Figure 4*), which demonstrated the relationship between the aneurysm and the adjacent vessel wall. A second guidewire was directed into the aneurysm sac using a double-lumen catheter and exchanged for a microcatheter. A 3.0 × 20 mm perfusion balloon was inflated across the aneurysm neck to maintain distal flow and prevent coil migration. Ten fibred platinum coils (20 × 500 mm) were deployed into the aneurysm cavity through a microcatheter positioned directly from the neck into the aneurysm. Angiography during the procedure confirmed contrast stasis and progressive thrombosis of the aneurysmal cavity (*Figure 5*). Final angiography showed complete exclusion of the aneurysm with preserved distal LAD perfusion. There were no procedural complications. IVUS confirmed intact vessel integrity without dissection or thrombus.

Echocardiography on postoperative day 1 confirmed the absence of flow within the aneurysm (*Figure 2B*). The patient remained stable post-procedure. She was discharged on single antiplatelet therapy (aspirin 100 mg daily). Follow-up angiography at 6 months and echocardiography at 1, 2, and 3 years showed sustained aneurysm occlusion and stable clinical status. The RCA-PA fistula was small and demonstrated only trivial shunt flow without chamber enlargement. On the basis of previous observational studies of small coronary-pulmonary fistulas, conservative management was considered appropriate.

## Discussion

This case illustrates several teaching points, particularly because the coexistence of a giant CAA and dual coronary-CPF is extremely rare, and current guidelines provide no specific recommendations for such anatomically complex presentations.

First, despite being asymptomatic, the aneurysm’s size (43 mm) and growth over 10 years indicated a high rupture risk,^4^ a progression suggesting structural instability. Although the shunt was mild (Qp/Qs: 1.15, calculated using the Fick principle during right heart catheterization), the persistent fistulous drainage from the LAD into the pulmonary artery likely generated chronic haemodynamic shear stress, promoting aneurysm expansion. Longitudinal monitoring with TTE proved essential in detecting this interval growth and reassessing the rupture risk.

Although current guidelines do not define a clear size threshold for intervention in asymptomatic CAAs,^5–7^ several reports indicate that most ruptured aneurysms exceeded 30 mm in diameter.^8^ The combination of giant size, interval enlargement, and high-flow fistulous communication constituted a high-risk anatomical scenario in this elderly patient. For these reasons, prophylactic intervention was deemed warranted to prevent potentially fatal rupture.

Regarding the additional fistula from the RCA, we elected conservative management. Unlike the LAD lesion, the RCA-PA fistula was not associated with a giant aneurysm. Since the fistula itself was not considered a therapeutic target given the mild shunt flow (Qp/Qs 1.15), we elected conservative management. In elderly frail patients, observational studies support a selective strategy in which only haemodynamically or structurally significant fistulas are treated. Given this patient’s anatomy and goals, focusing on exclusion of the rupture-prone LAD aneurysm while avoiding unnecessary procedural risk was considered safest.

Second, although surgical resection is regarded as a definitive treatment,^9^ posed significant risks in this frail patient with multiple comorbidities. We also considered percutaneous covered stent implantation, which has been used in other reported cases.^10^ However, in our patient, the aneurysm's large size (43 mm), mid-LAD location, and significant vessel diameter mismatch (proximal LAD approximately 5 mm, distal LAD approximately 2 mm) raised major concerns (*Figure 4*). The tapered landing zones likely resulted in poor stent apposition, increasing the risk of residual flow or stent thrombosis. These anatomic and procedural limitations rendered covered stenting unsuitable. Coil embolization was therefore selected as the optimal strategy.

The use of a perfusion balloon effectively prevented coil migration and preserved distal LAD perfusion, highlighting its importance in such interventions. At the 3-year follow-up, the patient remained free of ischaemic or haemorrhagic complications, with durable exclusion of the aneurysm. Noninvasive follow-up imaging, such as echocardiography, remains essential to confirm long-term thrombosis and exclude recanalization. Another key point is the advantage of avoiding stent implantation in the native LAD artery. This strategy minimized the risk of in-stent restenosis and eliminated the need for long-term dual antiplatelet therapy, which is particularly beneficial in this elderly patient considered at high bleeding risk (HBR).

## Conclusion

This case demonstrates coil embolization, guided by IVUS and balloon assistance, is a feasible treatment for giant CAAs with CPFs in high-risk elderly patients, offering durable outcomes and minimal invasiveness.

## Follow-up and outcomes

The patient has been followed clinically for 3 years post-procedure. She has remained asymptomatic without recurrence of the aneurysm or fistula flow. Echocardiography at 1, 2, and 3 years confirmed continued thrombosis of the aneurysm. There has been no evidence of ischaemia, arrhythmia, or heart failure. Aspirin monotherapy has been continued without bleeding complications. No additional intervention has been required.

## Lead author biography


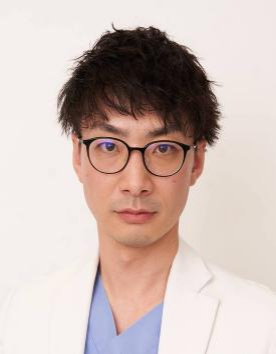
Dr Soichiro Shirabe, MD, is a cardiologist at Kyushu University Hospital, Japan. His clinical focus includes coronary artery disease and structural heart interventions. He has extensive experience in percutaneous coronary intervention (PCI), including complex cases such as aneurysmal lesions and arteriovenous fistulas. His research interests involve multimodal imaging, novel catheter-based therapies, and minimally invasive strategies for structural heart diseases. Dr. Shirabe is dedicated to improving outcomes in high-risk cardiovascular patients through individualized treatment approaches and evidence-based care.

## Data Availability

Given a reasonable request to disclose the patient's information, we can provide the patient's information with her anonymity.
